# A global dataset of pandemic- and epidemic-prone disease outbreaks

**DOI:** 10.1038/s41597-022-01797-2

**Published:** 2022-11-10

**Authors:** Juan Armando Torres Munguía, Florina Cristina Badarau, Luis Rodrigo Díaz Pavez, Inmaculada Martínez-Zarzoso, Konstantin M. Wacker

**Affiliations:** 1 Faculty of Economic Sciences, Georg-August-Universität Göttingen, Göttingen, Germany; 2 University of Bordeaux, CNRS, BSE, UMR 6060, F-33600 Pessac, France; 3 Department of Economics, University Jaume I, Castelló de la Plana, Spain; 4 Faculty of Economics and Business, University of Groningen, Groningen, The Netherlands

**Keywords:** Infectious diseases, Databases

## Abstract

This paper presents a new dataset of infectious disease outbreaks collected from the Disease Outbreak News and the Coronavirus Dashboard produced by the World Health Organization. The dataset contains information on 70 infectious diseases and 2227 public health events that occurred over the period from January 1996 to March 2022 in 233 countries and territories around the world. We illustrate the potential use of this dataset to the research community by analysing the spatial distribution of disease outbreaks. We find evidence of spatial clusters of high incidences (“hot spots”) in Africa, America, and Asia. This spatial analysis enables policymakers to identify the regions with the greatest likelihood of suffering from disease outbreaks and, taking into account their degree of preparedness and vulnerability, to develop policies that may help contain the spreading of future outbreaks. Further applications could focus on combining our data with other information sources to study, for instance, the link between environmental, globalization, and/or socioeconomic factors with disease outbreaks.

## Abstract (summary)

**Table:** 

Measurement(s)	Pandemic- and epidemic-prone disease outbreaks
Technology Type(s)	Text mining using R
Sample Characteristic - Organism	Disease outbreaks
Sample Characteristic - Environment	spatiotemporal region
Sample Characteristic - Location	Global

## Background & Summary

The COVID-19 pandemic has widely demonstrated the hazard that infectious diseases can pose to global public health and development. According to the latest available estimates from the World Health Organization (WHO), as of July 2022 it has been confirmed to have affected over 562 million people worldwide, having caused more than 6.3 million deaths^1^. However, COVID-19 is not the only infectious disease threatening the world. In 2019, the year before the first confirmed death from COVID-19, infectious diseases claimed more than 5.1 million lives, accounting for 14% of the 55.4 million deaths worldwide, approximately 4 million fewer than in 2000^2^.

Despite the decline in deaths from infectious diseases in the last two decades leading up to the COVID-19 pandemic, the world has also witnessed how disease outbreaks are emerging at unprecedented rates^3,4^. For instance, the outbreak of severe acute respiratory syndrome associated Coronavirus (SARS-CoV) in 2003^5^, the 2009–2010 influenza A(H1N1) pandemic^6^, the Middle East respiratory syndrome Coronavirus (MERS-CoV) outbreak in 2012^7^, the 2013–2016 west African Ebola virus disease epidemic^8^, and the 2015–2016 zika virus epidemic^9^; all spread in several countries across different continents, disproportionately impacting the most vulnerable communities^10,11^.

The increasing number of disease outbreaks has also lent impetus to a growing research interest on examining this phenomena^12^. However, this has gone hand-in-hand with a rising need for reliable open data from official sources. Existing datasets on the matter exclusively cover a limited number of infectious diseases^13–15^, or are specific to a population, country, or region^16–18^. Moreover, other datasets are based on unofficial information^19,20^, which may contain incorrect information or disinformation from false reports, or are not publicly available^21^, hampering their reuse and utilization.

The WHO, as part of its mandate, collects information about confirmed and potential public health events of concern in the world. Specifically for COVID-19, the WHO made available the Coronavirus Dashboard^1^. For the rest of diseases, the information is contained in the Disease Outbreak News (DONs)^22^. The information of the DONs is obtained from an integrated global system coordinated by the WHO. This information is based on epidemiological, clinical, and laboratory investigations conducted by the official public health authorities, institutions, and research networks of the WHO and its partners all over the world. However, since the DONs are not primary produced for statistical purposes, they are unstructured, with a format that makes it difficult to extract detailed information, and they do not make use of concepts and definitions that conform to international standards. Therefore, to create a database that is statistically sound for research purposes, we first processed the information from the DONs and then merged it with the Coronavirus Dashboard data. This data creation process is described in the Methods section.

Our final dataset contains information on 2227 disease outbreaks which occurred over the period from January 1996 to March 2022. In comparison with existing data on the matter^13–21^, our dataset provides five key advantages. First, a wide geographic coverage of 233 countries and territories around the world. Second, an extensive coverage of 70 infection diseases. Third, the utilization of standardized concepts and definitions, for which we used the codes of the International Standard Organization for countries and territories (ISO-3166)^23^, and the tenth revision of the International Statistical Classification of Diseases and related Health Problems (ICD-10)^24^. This allows the users to flexibly obtain information for a specific region, country, year, or disease. Fourth, for transparency, replicability, and reproducibility purposes, we make the data, metadata, and the code to create these data publicly available from Figshare^25^ both in human- and machine-readable formats (HTML, R, csv), which facilitates the reuse of the information. Moreover, by re-running the code the user can automatically extract more recent DONs from the website to keep the database updated. Finally, our data are interoperable, i.e. they can be easily integrated with other data by using the country code, the year, and/or the disease code as key variable(s) matching observations between datasets.

To illustrate the potential use of our dataset to the research community we apply an exploratory spatial data analysis (ESDA) in the Usage Notes. Our results show that outbreaks tend to be spatially clustered. We find evidence of the existence of spatial clusters of high incidences (“hot spots”) in Africa, America, and Asia. This spatial analysis enables policymakers to identify the countries with the greatest likelihood of suffering from disease outbreaks and, taking into account their degree of preparedness^26^ and vulnerability^27^, to develop policies that may help contain the spreading of future outbreaks^28^. Further research using this database may combine this information with other sources to identify the factors associated with the exposure of countries to pandemic- and epidemic-prone disease outbreaks^29,30^.

## Methods

### Data sources

Our data sources are the DONs and the Coronavirus Dashboard produced by the WHO^22^. This information is issued for general distribution under the Creative Commons Attribution-NonCommercial-ShareAlike 3.0 Intergovernmental Organization (CC BY-NC-SA 3.0 IGO) license, which enables users to freely copy, reproduce, reprint, distribute, translate, and adapt the WHO materials for non-commercial purposes.

The information from the DONs is available at www.who.int/emergencies/disease-outbreak-news and include all the reports on confirmed acute public health events or potential events of concern that occurred since 1996. Specifically, the DONs incorporate events of:

- Unknown cause but having a significant or potential health concern that may affect international travel or trade.
- A known cause with a demonstrated ability to produce a serious public health impact and spread internationally.
- High public concern potentially leading to disruption of required public health interventions or could disrupt international travel or trade.

Given the relevance of COVID-19, the WHO developed the Coronavirus Dashboard to provide official daily counts of COVID-19 cases, deaths, and vaccines^1^. The Coronavirus Dashboard is available at https://covid19.who.int/ and presents information as reported by official public health authorities of the countries and territories in the world.

### Data collection and integration processes

**Fig. 1:**
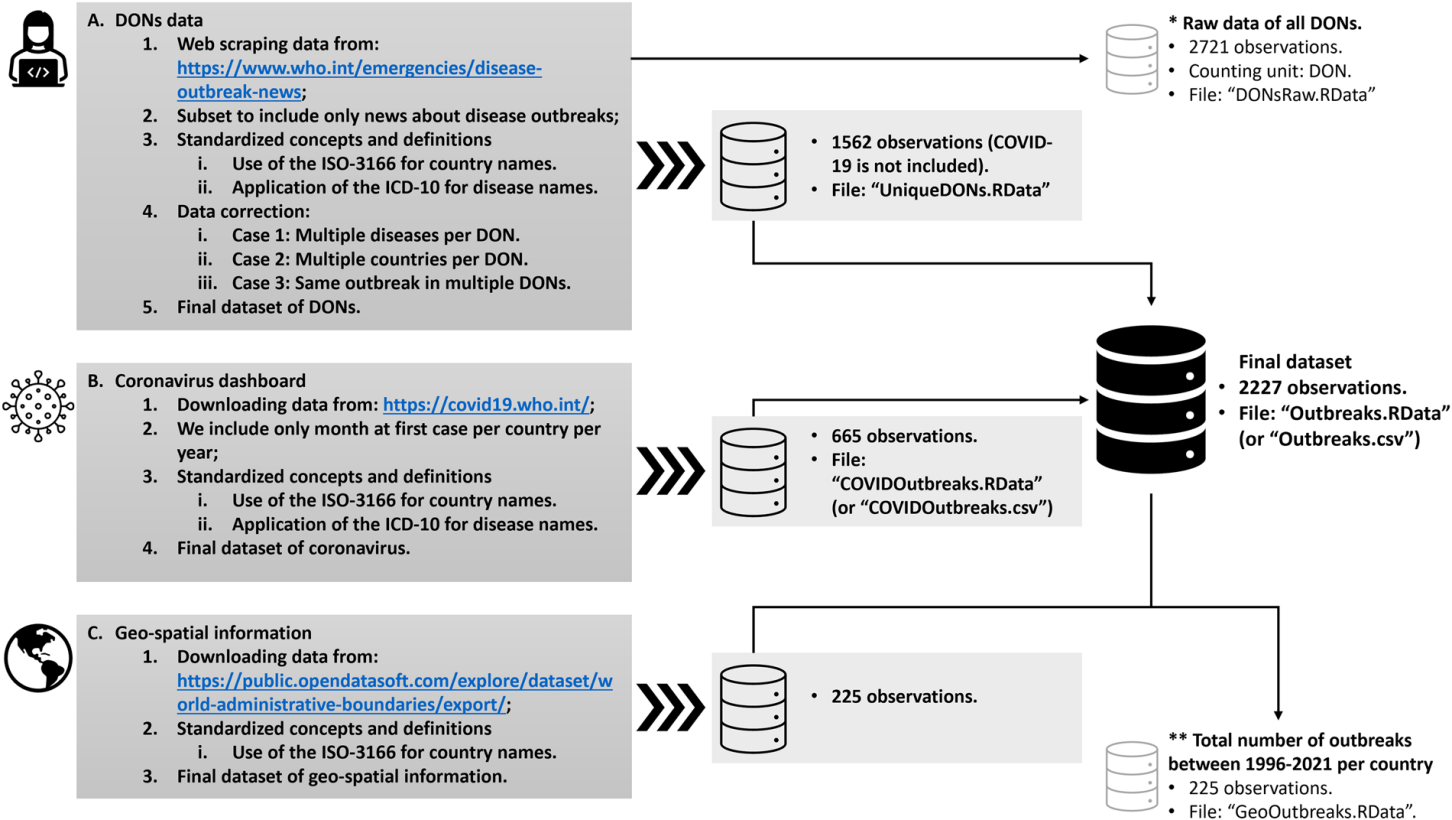
Schematic overview of the data collection and integration processes. (**A**) Data collection and database of the DONs; (**B**) Data collection and database of the Coronavirus Dashboard; (**C**) Geo-spatial information.

Figure 1 provides a schematic overview of the data collection and integration processes to create our dataset. These processes are described in detail below. The code to replicate all these steps, from web scraping to the creation of the final dataset, is found in the file “*DONs.R*”. This code, together with the databases have been made available from Figshare^25^. The permission to extract all the information from the WHO webpage and redistribute the resulting dataset under an open data license assigned to our Figshare was authorized under request 388191 submitted through WHO’s online platform.

In stage (**A**) of Fig. 1, we collected 2721 DONs from the WHO webpage. This collection was carried out by developing a code in R to automatically extract the information of the DONs. The oldest DON is related to cholera outbreaks reported on 22 January 1996 in Cabo Verde, Côte d’Ivoire, the Islamic Republic of Iran, Iraq, and Senegal. The most recent DON that is covered in our dataset refers to an outbreak of yellow fever in Kenya and was registered on 25 March 2022. To this dataset we added a column to uniquely identify each of the DONs. See file “*DONsRaw.RData*” for a raw dataset of the 2721 DONs.

Once the 2721 DONs were collected, we subset them to exclusively include those events related to the occurrence of disease outbreaks. In particular, we deleted DONs reporting other types of events, such as meetings of international experts (see DON0133), recommendations on treatments (DON0153), or travel requirements (DON0107 and DON0198).

To ensure the utilization of standardized concepts and definitions, we used the official short country names in English according to the ISO-3166^23^, and the ICD-10^31^.

The resulting dataset has still three recording issues that need to be tackled. First, some observations refer to multiple outbreaks. Second, some DONs inform about disease outbreaks happening in more than one country. Third, there are DONs registering the same outbreak multiple times due to situation updates. To resolve these issues, we proceed as follows. For DONs reporting more than one disease outbreak (for instance, DON0065 on influenza and malaria in Ghana, or DON1094 on chikungunya and dengue in the southwest Indian Ocean) and/or reporting more than one country (e.g., DON1540 about an outbreak of polio in Angola and the Democratic Republic of the Congo, or DON0617 on a meningococcal disease outbreak in the Great Lakes area) we replicate the DON as many times as diseases (or countries) are reported. For instance, DON0617 informs of an outbreak that occurred in Burundi, Rwanda, and Tanzania (Great Lakes area). Therefore, we registered this DON three times, i.e. one observation for each country.

Finally, to avoid multiplicity issues we deleted all the DONs registering the information on the same disease for the same country more than once in a given calendar year. For variants or mutations of viruses, as in the case of the avian influenza and its variants A(H1N1), A(H1N2), A(H5N1), A(H3N2), among others, all of them are considered as the same disease, i.e. influenza due to identified zoonotic or pandemic influenza virus. In this way, we ensured to have only one observation per disease, country and year. In other words, our unit of analysis in the database is an outbreak that happens when a country has at least one case of a specific disease during a particular year. Thus, a specific country cannot have two outbreaks related to the same disease in the same year but can have more than one outbreak of different diseases in the same year. Moreover, a country can only have more than one outbreak of the same disease if and only if they refer to different years. The resulting dataset consists of 1562 observations with unique outbreaks, covering a total of 217 countries and territories, and 69 different infectious diseases occurred over the period from January 1996 to March 2022. See file “*UniqueDONs.RData*”.

Since outbreaks related to COVID-19 are not included in the dataset of the DONs, we proceed to extract this information from the Coronavirus Dashboard, as shown in stage (**B**) of Fig. 1. In particular, we dichotomized the information on the cases per country per year to assign a one if the country had at least one case of Coronavirus, and zero otherwise. For standardization purposes, we used as in stage (A) the official short country names in English by the ISO-3166^23^, and the ICD-10^31^. The resulting dataset of Coronavirus outbreaks consists of 665 observations which occurred since 2020 and until 31^st^ March 2022, covering a total of 224 countries and territories. See file “*COVIDOutbreaks.RData*”.

The standardization of the country names allows us to use the country code as a key variable to merge the data of Coronavirus outbreaks with the outbreaks’ dataset of the DONs. The resulting data from merging these two datasets consist of 2227 observations of unique outbreaks of 70 different infectious diseases that occurred in a total of 233 countries and territories from 1996 until March 2022. See file “*Outbreaks.RData*”.

For the spatial analysis shown in the Usage Notes, we used in stage **C)** the dataset “*World Administrative Boundaries - Countries and Territories*”^32^ by the Société OPENDATASOFT available at https://public.opendatasoft.com/explore/dataset/world-administrative-boundaries/export/. This dataset is a shapefile with geographic information about the administrative boundaries of countries as well as non-sovereign territories and is published under the Open Government Licence v3.0. We added this geo-spatial information to the dataset with 2227 unique disease outbreaks by using as key variable the alpha-3 country code from the ISO 3166. See the code to create this dataset and replicate the analysis in file “*ESDAoutbreaks.R*” and the final dataset in file “*GeoOutbreaks.RData*”.

### Data management

To enhance the transparency, reproducibility, and reusability of our datasets for scientific purposes we follow the FAIR (Findability, Accessibility, Interoperability, and Reusability) guiding principles for scientific data management and stewardship^33^.

In particular, we addressed these principles by adding the following attributes to our database. First, we assigned a Digital Object Identifier (DOI) from Figshare^25^. This DOI is a globally unique and persistent identifier that makes the data findable. Second, in order to make the information accessible, this DOI is linked to a repository where users can freely retrieve the data files, documentation, and code. Third, to make the datasets interoperable we use terminologies and vocabularies that are commonly used in the field of epidemiology by using the ICD-10^31^ and the ISO-3166^23^. To document the database, metadata on Figshare uses the Fields of Research schema. Moreover, the datasets are available both in human- and machine-readable formats (HTML, R, csv) and thus, users can easily interact with the resources. Finally, in order to ensure reusability, we also include a metadata describing the variables in the datasets and we release the database with a Creative Commons Attribution 4.0 International (CC BY 4.0), which allows the user to share, copy, and redistribute the material in any medium or format, and to adapt, remix, transform, and build upon the material for any purpose.

## Data Records

The compiled database contains datasets and code in R language, which have been made available from Figshare^25^. Following the Fields of Research metadata schema from Figshare, the following files can be found in the repository:

1. “DONs.R”
  Categories: Statistics, Epidemiology, Diseases
  Item type: Software
  Keywords: Disease Outbreak News (DONs), the Coronavirus Dashboard, World Health Organization (WHO), Global database, International Statistical Classification of Diseases and related Health Problems, ISO 3166.
  Description: R-code to replicate the extraction of data from the Disease Outbreak News (DONs), available at www.who.int/emergencies/disease-outbreak-news, and the Coronavirus Dashboard, available at https://covid19.who.int/, produced by the World Health Organization (WHO). In addition, this code includes the integration, data correction, and use of standard names and definitions from the ISO 3166 and the International Statistical Classification of Diseases and related Health Problems.
2. “DONsRaw.RData” (or alternatively “DONsRaw.csv)
  Categories: Statistics, Epidemiology, Diseases
  Item type: Dataset
  Keywords: Disease Outbreak News (DONs), the Coronavirus Dashboard, World Health Organization (WHO), Global database.
  Description: Dataset containing the raw data from 2721 DONs as extracted from www.who.int/emergencies/disease-outbreak-news. The dataset presents information from 1996 to March 2022 (last DON was registered on 25 March 2022).
  Data records:
  “**ID**”: DON unique identifier.
  “**Description**”: Name of the DON as extracted from www.who.int/emergencies/disease-outbreak-news.
  “**Date**”: Date of registry of the DON.
  “**Link**”: Link to the website to find more information on the DON.
3. “UniqueDONs.RData” (or alternatively “UniqueDONs.csv”)
  Categories: Statistics, Epidemiology, Diseases
  Item type: Dataset
  Keywords: Disease Outbreak News (DONs), the Coronavirus Dashboard, World Health Organization (WHO), Global database.
  Description: Dataset containing 1562 observations with unique outbreaks, covering a total of 217 countries and territories, and 69 different infectious diseases. A unique outbreak happens when a country has at least one case of a specific disease during a given year. Thus, a specific country cannot have two outbreaks related to the same disease in the same year (but can have more than one outbreak of different diseases in the same year). Moreover, a country can only have more than one outbreaks of the same disease, if and only if they refer to different years.
  Data records:
  “**Country**”: Name of the country where the outbreak occurred.
  “**iso2**”: Alpha-2 country code from the ISO 3166.
  “**iso3**”: Alpha-3 country code from the ISO 3166.
  “**Year**”: Year of occurrence of the outbreak.
  “**icd10n**”: Name of the type of disease according to the ICD-10.
  “**icd103n**”: Name of the subtype of disease according to the ICD-10.
  “**icd104n**”: Name of the disease according to the ICD-10.
  “**icd10c**”: Code of the name of the type of disease according to the ICD-10.
  “**icd103c**”: Code of the name of the subtype of disease according to the ICD-10.
  “**icd104c**”: Code of the name of the disease according to the ICD-10.
  “**icd11c1**”: Code of the name of the type of disease according to the ICD-11.
  “**icd11c2**”: Code of the name of the subtype of disease according to the ICD-11.
  “**icd11c3**”: Code of the name of the disease according to the ICD-11.
  “**icd11l1**”: Name of the type of disease according to the ICD-11.
  “**icd11l2**”: Name of the subtype of disease according to the ICD-11.
  “**icd11l3**”: Name of the disease according to the ICD-11.
  “**Disease**”: Name of the disease.
  “**DONs**”: List of DONs reporting the outbreak.
  “**Definition**”: Definition of the disease according to the ICD-11.
4. “COVIDOutbreaks.RData” (or alternatively “COVIDOutbreaks.csv”)
  Categories: Statistics, Epidemiology, Diseases
  Item type: Dataset
  Keywords: Disease Outbreak News (DONs), the Coronavirus Dashboard, World Health Organization (WHO), Global database.
  Description: Dataset containing 665 observations occurred since 2020 and until March 2022, covering a total of 224 countries and territories in which cases of COVID-19 were reported. In particular, we dichotomized the information on the cases per country per year to assign a one if the country had at least one case of Coronavirus, and zero otherwise.
  Data records:
  “**Country**”: Name of the country where the outbreak occurred.
  “**iso2**”: Alpha-2 country code from the ISO 3166.
  “**iso3**”: Alpha-3 country code from the ISO 3166.
  “**Year**”: Year of occurrence of the outbreak.
  “**icd10n**”: Name of the type of disease according to the ICD-10.
  “**icd103n**”: Name of the subtype of disease according to the ICD-10.
  “**icd104n**”: Name of the disease according to the ICD-10.
  “**icd10c**”: Code of the name of the type of disease according to the ICD-10.
  “**icd103c**”: Code of the name of the subtype of disease according to the ICD-10.
  “**icd104c**”: Code of the name of the disease according to the ICD-10.
  “**icd11c1**”: Code of the name of the type of disease according to the ICD-11.
  “**icd11c2**”: Code of the name of the subtype of disease according to the ICD-11.
  “**icd11c3**”: Code of the name of the disease according to the ICD-11.
  “**icd11l1**”: Name of the type of disease according to the ICD-11.
  “**icd11l2**”: Name of the subtype of disease according to the ICD-11.
  “**icd11l3**”: Name of the disease according to the ICD-11.
  “**Disease**”: Name of the disease.
  “**DONs**”: Coronavirus Dashboard.
  “**Definition**”: Definition of the disease according to the ICD-11.
5. “Outbreaks.RData” (or alternatively “Outbreaks.csv”)
  Categories: Statistics, Epidemiology, Diseases
  Item type: Dataset
  Keywords: Disease Outbreak News (DONs), the Coronavirus Dashboard, World Health Organization (WHO), Global database.
  Description: Dataset containing 2227 observations (unique disease outbreaks), occurred in a total of 233 countries and territories from 1996 and until March 2022, and associated to 70 different infectious diseases. A unique outbreak happens when a country has at least one case of a specific disease during a given year.
  Data records:
  “**Country**”: Name of the country where the outbreak occurred.
  “**iso2**”: Alpha-2 country code from the ISO 3166.
  “**iso3**”: Alpha-3 country code from the ISO 3166.
  “**Year**”: Year of occurrence of the outbreak.
  “**icd10n**”: Name of the type of disease according to the ICD-10.
  “**icd103n**”: Name of the subtype of disease according to the ICD-10.
  “**icd104n**”: Name of the disease according to the ICD-10.
  “**icd10c**”: Code of the name of the type of disease according to the ICD-10.
  “**icd103c**”: Code of the name of the subtype of disease according to the ICD-10.
  “**icd104c**”: Code of the name of the disease according to the ICD-10.
  “**icd11c1**”: Code of the name of the type of disease according to the ICD-11.
  “**icd11c2**”: Code of the name of the subtype of disease according to the ICD-11.
  “**icd11c3**”: Code of the name of the disease according to the ICD-11.
  “**icd11l1**”: Name of the type of disease according to the ICD-11.
  “**icd11l2**”: Name of the subtype of disease according to the ICD-11.
  “**icd11l3**”: Name of the disease according to the ICD-11.
  “**Disease**”: Name of the disease.
  “**DONs**”: DONs reporting the outbreak. For the case of the Coronavirus, the source is the Coronavirus Dashboard.
  “**Definition**”: Definition of the disease according to the ICD-11.
6. “ESDAoutbreaks.R”
  Categories: Statistics, Epidemiology, Diseases
  Item type: Software
  Keywords: Disease Outbreak News (DONs), the Coronavirus Dashboard, World Health Organization (WHO), Global database, Exploratory spatial data analysis (ESDA).
  Description: R-code to replicate the exploratory spatial data analysis (ESDA).
7. “GeoOutbreaks.RData”

Categories: Statistics, Epidemiology, Diseases

Item type: Dataset

Keywords: Disease Outbreak News (DONs), the Coronavirus Dashboard, World Health Organization (WHO), Global database, Geo-spatial information.

Description: Dataset containing the total number of unique disease outbreaks per country with geo-spatial information.

Data records:

“**iso3**”: Alpha-3 country code from the ISO 3166.

“**region**”: Region of the country.

“**continent**”: Continent of the country.

“**name**”: Name of the country where the outbreak occurred.

“**french**_**shor**”: Name of the country in French.

“**freq**”: Total frequency of disease outbreaks.

“**geometry**”: Geographic coordinates of the country.

## Technical Validation

**Fig. 2:**
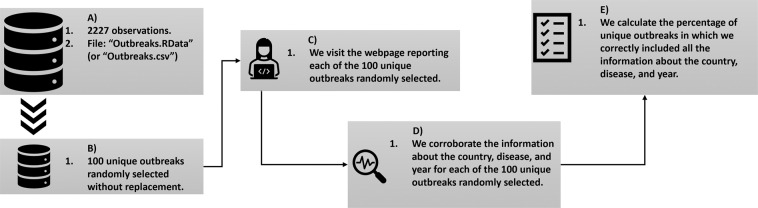
The schematic overview of the technical validation processes is illustrated in five steps: (**A**) Database with 2227 unique outbreaks (Outbreaks.RData); (**B**) Random selection of 100 unique outbreaks; (**C**) Information on the web about the DONs; (**D**) Corroboration of the information; (**E**) Validation results.

The process of technical validation is described in a schematic form in Fig. 2.

In step (A) we uploaded the final dataset in R. From it, we randomly selected a sample of 100 observations from the 2227 disease outbreaks (B). Then, we used the information in the column DONs, that includes the unique identifier of the DONs reporting that outbreak, to manually check the information by accessing to the corresponding link of the WHO where the DON is described (C). Three attributes were observed: information about the country, disease, and year. After validating all the information (D), we obtained a 98% of correctness for the information about the country (we initially wrote “Tanzania”, instead of “Tanzania United Republic of”, and “Moldova”, instead of “Moldova Republic of”), 99% for disease (we erroneously wrote in column icd104n “Dengue fever, unspecified”, instead of “Dengue, unspecified”), and 100% for year (E). The three cases with errors were corrected in the original code producing the data (“DONs.R”) and we rerun the code to obtain a corrected version of the dataset.

## Usage Notes

The format of our dataset allows the users to flexibly extract and manipulate the information, as well as put it together with other datasets when using the country code from the ISO-3166 or the disease code from the ICD-10.

Target applications of the database presented in this paper should focus on combining our datasets with variables from other sources to study, for instance, the link between spatial, temporal, environmental, globalization, or socioeconomic factors with the exposure of countries to disease outbreaks. Moreover, researchers may be interested in extracting a subset of data to analyze a specific country or disease and integrate it with related country-specific or disease-specific information to describe more in detail the outbreaks.

As an example, we apply an exploratory spatial data analysis (ESDA) to the total frequency of outbreaks by country (or territory) between 1996 and 2021 (2022 is not included, given that only the first three months of the year are available). The code to create the dataset with geographic information and replicate this analysis can be found in the file “*ESDAOutbreaks.R*”.

### Empirical strategy

The goal of the ESDA applied to our data is to detect significant spatial patterns in disease outbreaks. The methodology used consists of the following three steps:

1. **Global Moran’s I estimation**. Under the null hypothesis that our recorded disease outbreaks are randomly distributed in space, this method evaluates the Moran’s I statistic for spatial autocorrelation, i.e. the correlation between the frequency of outbreaks across the geographical space. The formula to calculate the Moran’s I statistics is expressed as follows:
  $$I=\frac{n\sum {\sum }_{{w}_{ij}}\left({x}_{i}-\bar{x}\right)\left({x}_{j}-\bar{x}\right)}{\sum {\sum }_{{w}_{ij}}\sum {\left({x}_{i}-\bar{x}\right)}^{2}}$$
  where *x*_*i*_, *x*_*j*_ are the observed frequency of outbreaks for countries (or territories) *i, j*; $\bar{x}$ is the average of the frequency of outbreaks over the *n* countries (or territories); *w*_*ij*_ denotes spatial weights. To estimate the global Moran’s I we considered the “Queen neighborhood structure”, i.e. two or more countries are considered to be contiguous if their respective polygons meet at least at a single point^34^. To each neighboring relationship we assign spatial weights considering the variance-stabilizing approach^35^, given that this approach moderates the impact of having countries with a very different number of neighbor links. Finally, we adjust the number of observations since some countries do not have neighbors, as is the case for islands.
2. **Local Moran’s I estimation**. When the calculated global Moran’s I is positive and statistically significant, it can be decomposed to obtain a measure for local spatial autocorrelation. The main idea behind the local Moran’s I is to provide an indication of the extent to which there is significant clustering of similar number of outbreaks around each country^36^.
3. **Cluster characterization**. In cases in which the local Moran’s I indicated the existence of non-randomly distributed data, we proceed to classify it into two different types of patterns based on the similarity or dissimilarity with their neighboring countries. On the one hand, we identified clusters, i.e. groups of neighboring countries with similar outbreak frequencies. Clusters with a higher concentration of outbreaks -compared to the expected number of outbreaks under random distribution- are called *High-High*. By contrast, clusters with a lower concentration of outbreaks relative to the expected number of outbreaks under random distribution are called *Low-Low*. On the other hand, we identified a group of countries with outbreak frequencies very different to their neighbors. If the cluster has a country with a high frequency of outbreaks and their neighbors have a significantly lower number of outbreaks, we label it as a *High-Low* cluster. If the cluster has a country with a low frequency of outbreaks and their neighbors have a significantly larger number of outbreaks, we refer to it as a *Low-High* cluster.

## Results

**Fig. 3:**
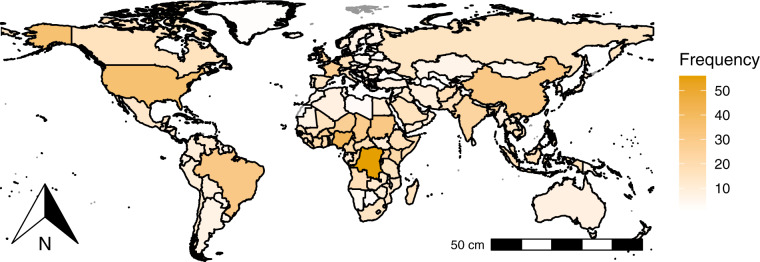
Map of infectious disease outbreaks.

Figure 3 depicts the geographical distribution of the outbreaks data. By continents, most of the outbreaks occurred in African countries (39.2%). Next in the ranking is Asia (23.5%), then America (17.2%), followed by Europe (16.8%), and Oceania (3.4%).

**Fig. 4:**
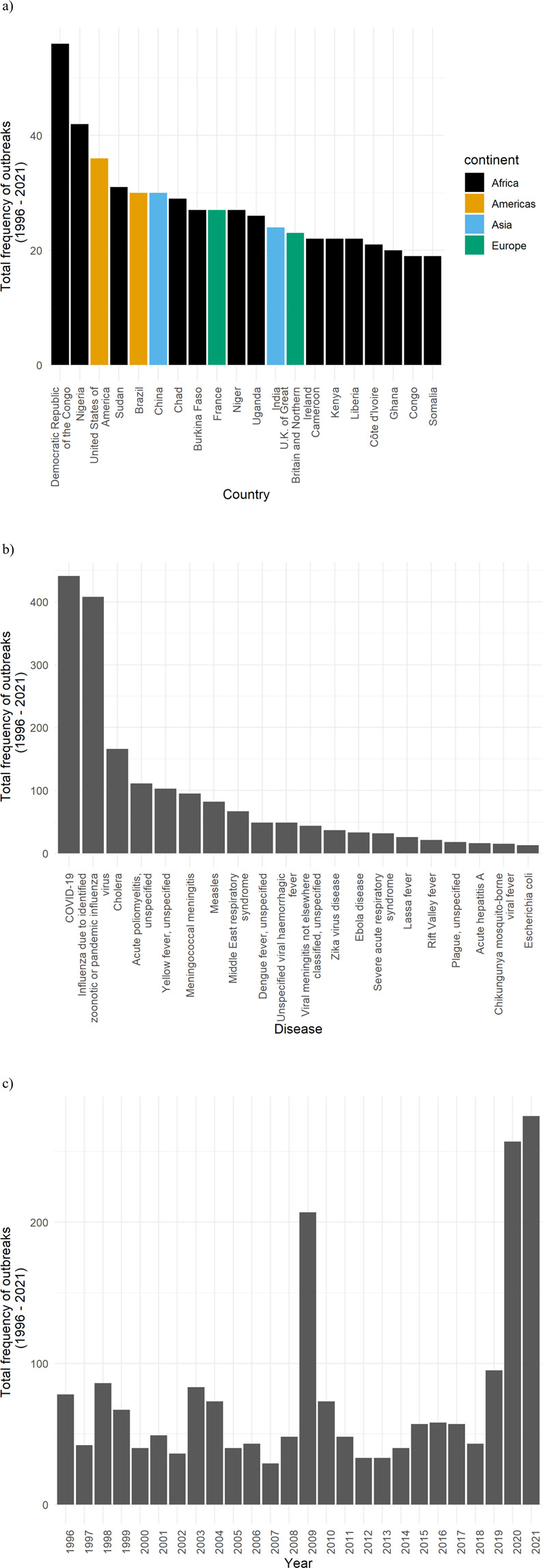
Number of outbreaks by country, disease and year. (**a**) Top 20 countries with the highest number of outbreaks; (**b**) Number of outbreaks by disease; (**c**) Number of outbreaks by year.

The countries with the highest number of disease outbreaks are the Democratic Republic of the Congo, Nigeria, United States of America, Sudan, Brazil, and China. The five diseases with the highest number of outbreaks are COVID-19, pandemic influenza virus, classical cholera, acute poliomyelitis, and yellow fever, in this order. The year reaching the highest record of unique outbreaks is 2021, followed by 2020, 2009, 2019, 1998, 2003, and 1996. The distributions of the outbreaks per country (a), disease (b), and year (c) are shown in Fig. 4.

### Global moran’s I

The spatial pattern of autocorrelation is statistically distinguishable from a random distribution (Moran’s I = 0.34, p < 0.001; see Table 1). Not surprisingly, the positive value of the statistic suggests that similar values, highs, or lows, are spatially clustered.

**Table 1:** Global Moran’s I statistic for total frequency of disease outbreaks (1996–2021).

Global Moran’s I statistic	**0.336**
p-value	**<0.001**

**Fig. 5:**
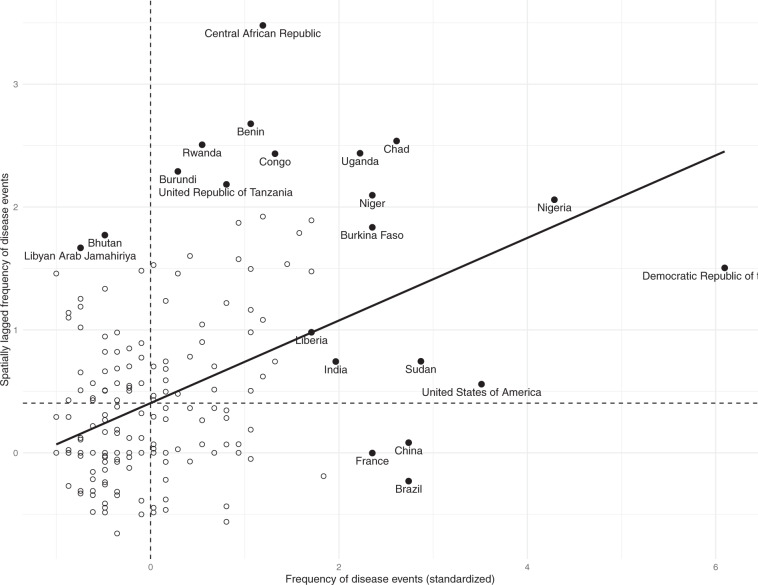
Moran scatterplot of the frequency of outbreaks.

Another way to examine the global autocorrelation is to create a scatterplot with the frequency of outbreaks of the countries (x-axis) and the spatially weighted sum of outbreaks of their respective neighbors (y-axis) and observe whether the data follows a significant relationship. Figure 5, known as Moran scatterplot, provides a visual representation of the four clustering categories previously mentioned, namely *High-High*, *High-Low*, *Low-High*, and *Low-Low*. Since the plot is centered at zero, all points to the right (or above zero) are associated to *High* values. Similarly, all the points to the left (or below zero) refer to *Low* values. Thus, each quadrant is linked to one of the four clustering patterns. For instance, the upper right quadrant corresponds to a positive autocorrelation, i.e. similar values are observed at neighboring countries^37^. In Fig. 5 we observe a regression line with a positive slope, indicating that countries with high frequency of outbreaks are generally neighbored by countries also having a high number of outbreaks, which corroborates the findings from the Global Moran’s I in Table 1.

### Local Moran’s I

**Fig. 6:**
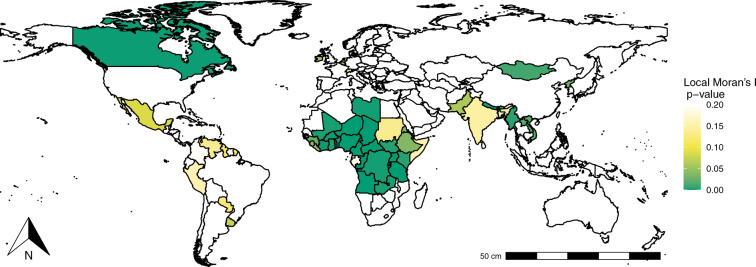
Local significance map.

Given that the hypothesis of randomly distributed outbreaks in space is rejected, the global indicator can be decomposed by country to obtain the local autocorrelation. To this aim, we create a local significance map to visualize the statistical significance at which each country is regarded as having a relevant contribution to the global autocorrelation^37^. Figure 6 depicts the local significance map, in which each country is colored according to their significance level (the greener the most significant). Significant clusters seem to be found in three regions, namely North America, Africa, and South and South-East Asia.

### Cluster characterization

**Fig. 7:**
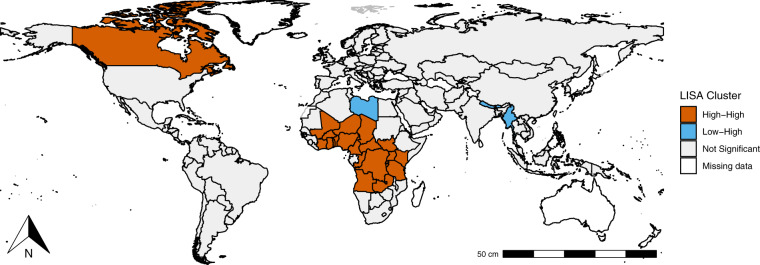
LISA clusters.

After obtaining the significance for each country, we examine the local indicators of spatial autocorrelation (LISA). This analysis allows us to identify which countries have a significant relationship with its surroundings, and the type of clustering they are conforming. In Fig. 7 we depict the significant clusters –using the 99% significance level– and by colors we indicate the type of clustering pattern they follow.

All in all, we find evidence on the existence of three clustering groups. First, in North America a *High-High* cluster is found in which Canada (16 outbreaks) and the United States of America (36) show a higher number of outbreaks in comparison to the expected number of outbreaks under a random distribution. This is mainly attributed to outbreaks related to influenza due to identified zoonotic or pandemic influenza virus. In particular, almost 23 percent of the total unique outbreaks of these two countries was related to this disease.

A second cluster is identified in South and South-East Asia. There, a *Low-High* pattern is constituted by countries such as Bhutan (5), Nepal (5), Macao (1), and Myanmar (8), which have a relatively low number of outbreaks but are neighboring with China (30) and India (24), that have a high frequency. In this cluster, we can also find a *High-High* pattern in Hong Kong (11 outbreaks), which also shares it border with China.

The third cluster identified is the largest one in the world and accounts for a total of 487 outbreaks between 1996 and 2021. It comprises 22 countries, namely, Angola, Benin, Burkina Faso, Burundi, Cameroon, Central African Republic, Chad, Congo, Democratic Republic of the Congo, Côte d’Ivoire, Ghana, Kenya, Libya, Mali, Niger, Nigeria, Rwanda, South Sudan, the United Republic of Tanzania, Togo, Uganda, and Zambia. According to our findings, there are two different clustering patterns in this region. There is a *Low-High* cluster formed by Libya (3 outbreaks), that is neighbored by Niger (27), Chad (29), and Sudan (31) that have a relatively large number of outbreaks. Moreover, a *High-High* pattern is integrated by a group of 21 contiguous countries recording a higher number of outbreaks relative to the expected number of outbreaks under a random distribution. Five infectious diseases are mainly explaining this cluster, namely classical cholera (almost 15 percent), COVID-19 (13.5 percent), acute poliomyelitis and meningococcal meningitis (close to 13 percent each), and yellow fever (about 10 percent).

These findings highlight the relevance of the geographical space in the study of disease outbreaks that, put in relation with other preparedness^26^ or vulnerability^27^ indexes, could help public health authorities and policy makers to design specific strategies targeted at regions or countries most affected, and to deploy resources in a cost-effective way, which would ameliorate the outbreak’s impact on mortality. Information about the geographical distribution of disease outbreaks may also be essential for national governments to advice individuals on whether to vaccinate before travel, for example. It can also help public health authorities to improve bio-surveillance and act for a better long-term preparedness in the most exposed countries to disease outbreaks.

Although the database presented significantly contributes to the study of global patterns in disease outbreaks, it has some limitations. We caution that studies using our data should be aware of two main issues when interpreting their results. First, information exclusively captures the occurrence of an outbreak associated to a particular disease during a given year in a country and not the intensity. This is an important contribution but does not reflect other relevant aspects in the study of epidemics, such as the number of cases or deaths associated to the outbreak, which are not available in the DONs. Second, information is only reported at the national level since no sub-national details are available. Future research could integrate our data with available mortality statistics by country and year and cause of death.

## Data Availability

Code in R language to replicate the database creation have been made available from Figshare^25^.
